# Epineural adipose‐derived stem cell injection in a sciatic rodent model

**DOI:** 10.1002/brb3.1027

**Published:** 2018-06-19

**Authors:** Elisabeth A. Kappos, Patricia Baenziger‐Sieber, Mathias Tremp, Patricia E. Engels, Sarah Thommen, Lima Sprenger, Robyn M. Benz, Dirk J. Schaefer, Stefan Schaeren, Daniel Felix Kalbermatten

**Affiliations:** ^1^ Department of Plastic, Reconstructive, Aesthetic and Hand Surgery University Hospital Basel Basel Switzerland; ^2^ Department of Neuropathology Institute of Pathology University Hospital Basel Basel Switzerland; ^3^ Basel Institute for Clinical Epidemiology and Biostatistics University Hospital Basel Basel Switzerland; ^4^ Department of Radiology University Hospital Basel Basel Switzerland; ^5^ Department of Spinal Surgery University Hospital of Basel Basel Switzerland

**Keywords:** adult stem cells, nerve compression syndromes, nerve regeneration, tissue engineering, treatment outcome

## Abstract

**Background:**

The aim was to evaluate the regenerative effect of epineural injection of rat ASCs (rASCs) in three different settings of acute and chronic compression in a rat sciatic nerve model.

**Methods:**

Acute compression (60 s) with a vessel clamp over a distance of 1 mm (group 1) or 10 mm (group 2), as well as chronic compression with a permanent remaining, nonabsorbable polymeric clip over a distance of 1 mm (group 3) was performed. Depending on the group, either 5 × 10^6^ rASCs or the same volume (25 μl) of culture medium (CM) was injected with a 30G needle in the epineurium at the time of compression. Outcome measures were functional gait evaluations, imaging analysis, histomorphometric analyses, and muscle weight.

**Results:**

The rats in group 2 had a better function than those with group 1 at one and especially at 2 weeks. After 4 weeks however, almost all rats were close to a normal function. There was a similar Muscle Weight Ratio (MWR) after 2 weeks in all groups, whereas after 4 weeks, the MWR in group 3 was lower compared with group 1 and 2. Histomorphometric analysis showed a better myelination in group 1 & 2 compared to group 3 after 4 weeks. ASCs have a beneficial effect on myelin thickness (G‐Ratio).

**Conclusions:**

We successfully evaluated the regenerative effect of epineural injection of rASCs in three different settings of acute and chronic compression. However, there were no significant differences in outcomes between the ASC‐treated groups and control groups.

## 1 INTRODUCTION

The outcome of peripheral nerve crush injury is often unsatisfying despite the intrinsic capability of peripheral nerve regeneration and a considerable progress in medical science over the past decades (Wood, Kemp, Weber, Borschel, & Gordon, 2011). The intrinsic regenerative process is with a maximum regrowth rate of 1 mm per day very slow, complicated with further problems as hyper‐ or hypoinnervations and also microsurgical interventions are rarely beneficial as the nerve‐continuity is preserved in crush injuries (Sta, Cappaert, Ramekers, Baas, & Wadman, 2014; Sunderland, 1947; Wood et al., 2011). Driven by the enormous clinical need of therapeutic alternatives, peripheral nerve regeneration has become an important research field of tissue engineering (Engels et al., 2013; Kappos et al., 2015; Tremp et al., 2015).

The interest in cell transplantation in the field of neurodegeneration and neuroregeneration increased tremendously in the last years. Mesenchymal stem cells (MSCs) are said to have a positive influence on peripheral nerve regeneration by several mechanisms, namely by trophic factor secretion, immune modulation, synthesis of extracellular matrix molecule, and stabilization of the microenvironment (Engels et al., 2013).

Among the various options of cell therapy, adipose‐derived stem cells (ASCs) distinguish positively by a great availability (Engels et al., 2013), high proliferation rates, the possibility of minimally invasive withdrawal and the characteristic multipotency (Papalia et al., 2013; Taha & Hedayati, 2010; Zuk et al., 2001, 2002).

Both degeneration and regeneration are studied on the basis of different morphological, behavioral, and electrophysiological techniques (Mazzer, Barbieri, Mazzer, & Fazan, 2008; Schiaveto de Souza, da Silva, & Del Bel, 2004). An improved behavioral outcome remains one of the most important evidence of functionality of axon regeneration after any therapy strategy and can be evaluated by walking track analysis (Chen et al., 2014; Pan et al., 2009).

The aim of this study was to evaluate the regenerative effect of an epineural injection of rat ASCs (rASCs) in three different settings of acute and chronic compression after 2 and 4 weeks in a rat sciatic nerve model.

## 2 MATERIALS AND METHODS

### 2.1 Rat ASC cultures and PKH26 labeling

Rat ASCs from the inguinal region were harvested following a well‐established protocol from our group (Kingham et al., 2007). For long‐term in vivo cell tracking, the cell membranes of rASCs were labeled by PKH26 red fluorescent cell linker (Sigma Aldrich, PKH26GL) according to the manufacturers’ recommendations. The labeled cells were afterward injected in one animal per group for each time point.

### 2.2 Animals and surgical procedures

Table 1 shows the three groups. For the sciatic nerve crush, 8‐week‐old female Sprague‐Dawley (SD) rats obtained from Harlan Laboratories underwent unilateral sciatic nerve crush. The ethical approval was obtained for all experiments by the local veterinary physician and the local ethical committee (No. 2672). All surgical procedures were performed under general anesthesia with isoflurane 2.5%. Prior to the surgery, Buprenorphine (Temgesic^®^, 2 ml Amp, 0.3 mg/ml, 0.1‐0.2 mg/kg BW) was administered.

**Table 1:** Trauma and treatment groups

Groups	Group 1: Acute short trauma (1 mm)	Group 2: Acute long trauma (10 mm)	Group 3: Chronic trauma (clip)
Treatment	Control	Control	Control
	ASCs	ASCs	ASCs

*Note*

ASCs: adipose‐derived stem cells.

Placed in prone position, the left hindquarter was shaved and disinfected using Betadine^®^, and the skin was incised parallel to the femur, and the sciatic nerve was approached as previously described (Kappos et al., 2015; Tremp et al., 2015). Then, the nerve was squeezed for one minute by using two different types of clamps (in order to squeeze the nerve over a distance of 1 mm (group 1), respectively 10 mm (group 2), just distally to the place where the small muscle branch sets aside from the sciatic nerve (about 1 cm distally to the spine). The squeezed area was subsequently marked at the proximal end with a single epineural suture (9/0 Nylon, Ethicon). In the chronic compression group (group 3), the equivalent segment (1 mm) of the nerve was clipped constantly by a nonabsorbable, polymeric clip (Weck^®^ Hem‐o‐lok^®^ Auto Endo5^®^).

Depending on the group, either 5 × 10^6^ rASCs in 25 μl or the same volume of CM (control) was injected with a 30G needle in the epineurium exactly there where the squeezing took place. Afterward, the cavity was rinsed using sterile NaCl 0.9% and then the muscles were readapted without using any sutures because they only have been divided and not cut. The skin was closed with absorbable single stitches (4/0 Vicryl, Ethicon). The closed wound was disinfected with Betadine^®^ to prevent any bacterial contamination. To avoid skin irritations, the sutures were removed after 10 days.

### 2.3 Behavioral analysis

In order to assess the recovery of motor function, a walking track analysis was performed using the CatWalk XT (Noldus Information Technology, the Netherlands). In every animal, three repeated measurements were carried out under constant conditions to record the steadiness of gait. The measurements took place preoperatively as control as well as postoperatively after 1, 2, and 4 weeks as previously described (Kappos et al., 2015). A sciatic functional index (SFI) of 0 is normal. A SFI of ‐ 100 indicates total impairment, such as would result from a complete transection of the sciatic nerve (Cheng et al., 1997; Kanaya, Firrell, Tsai, & Breidenbach, 1992).

### 2.4 MRT scanning

The clinical 3T MRI scanner has been shown useful to monitor the efficacy of cellular therapy over time (Tremp et al., 2015). MRI of the rats was performed immediately postmortem on a 3T MRI scanner (Magnetom Prisma, Siemens Healthcare GmbH, Erlangen/Germany) using a 16‐channel phased‐array wrist coil. PMDTI was performed with diffusion‐weighted spin echo single‐shot echo planar imaging (EPI) using the following parameters: TR/TE = 9,200/91 ms, averages = 2, field of view (FOV) = 120 × 100 mm, matrix size = 100 × 100, slice thickness = 4.0 mm, resulting in a voxel size of 1.2 × 1.2 × 3.0 mm, *b*‐values of 0 and 1,200 s/mm^2^, and 20 gradient directions. The data were postprocessed using MITK‐Diffusion as previously reported (Fritzsche et al., 2012). The sciatic nerve was identified on the *b* 1,200 s/mm^2^ image and the plane was adjusted to intersect the nerve perpendicular on the injured side. A region of interest (ROI) was positioned encompassing the nerve. From this ROI, the fractional anisotropy (FA) was extracted.

### 2.5 Muscle atrophy

After euthanasia, the left (experimental) and right (control) gastrocnemius muscles were excised and weighted with an analytical balance (Mettler‐Toledo, Switzerland). For each rat, a gastrocnemius muscle weight ratio (MWR) was calculated based on the wet muscle weight according to the following formula:

$$\frac{E}{C}=\frac{\text{weight experimental muscle}}{\text{weight control muscle}}$$

### 2.6 Morphometric analyses

For morphometric analyses, the sciatic nerves were harvested two and 4 weeks after the surgery from all groups. The proximal and the distal portion of the nerves were prepared for 1.5 μm semithin cross sections. Therefore, the nerve tissue was fixated in 2.5% glutaraldehyde for 2 hr followed by PBS overnight. After postfixation in 1% osmium tetroxide (OsO4) for 2 hr and following dehydration through a graded series of ethanol, the tissue was embedded in epoxy resin (Durcupan^®^). Afterward, 1.5 μm semithin sections were cut with glass knives (Ultramicrotome “Ultracut E”, Reichert‐Jung) and finally stained with 1% paraphenylenediamine.

The cross sections were analyzed under a light microscope (Olympus BX43, Japan), using a 10x and 40x magnification objective. With the help of ImageJ 1.48f nerve fibers of each sample were screened within representative fields for following parameters: Average axonal diameter (AD), fiber diameter (FD), and myelin thickness (MT). According to Yu et al (Yu, Liu, Ma, & Xiang, 2014; Yu et al., 2011), the myelination was estimated by the G‐ratio (=axon diameter/fiber diameter). Finally, the fiber density per mm^2^ could be calculated.

The middle portion of the nerves including the squeezed area were fixated overnight in 4% paraformaldehyde, dehydrated, and further processed for paraffin‐embedding. From these samples, 3 μm thin longitudinal sections were cut and stained with hematoxylin and eosin (HE) as well as with holmes–luxol (HL) following the house‐interne staining protocols. Images were also acquired under the light microscope.

All the morphometric analyses were carried out by an observer blinded to the experimental groups and localizations (proximal, distal). Blinding was achieved by numbering the nerve samples. Decoding took place after analyses were completed.

### 2.7 Statistical analysis

Data are presented as mean ± standard deviations (*SD*) or median and interquartile range (IQR) where appropriate from at least three animals per group and all experimental samples were conducted in triplicate. One‐way analysis of variance (ANOVA) test with corresponding post hoc tests (Bonferroni or Tukey test) were used where appropriate to determine statistical differences between the experimental groups. *p* < 0.05 was determined to be significant. Linear models were used to estimate the effect of treatment (ASC vs. control), time (four vs. 2 weeks post‐trauma), and the trauma (“long acute trauma” or “chronic trauma” versus “short acute trauma,” respectively) on Delta FA, MWR, and delta G‐Ratio. All statistical analyses were performed in R Version 3.2.3 (R Foundation for Statistical Computing, Vienna, Austria) (R Core Team, 2014).

## 3 RESULTS

### 3.1 Morphometric evaluation

**Figure 1:**
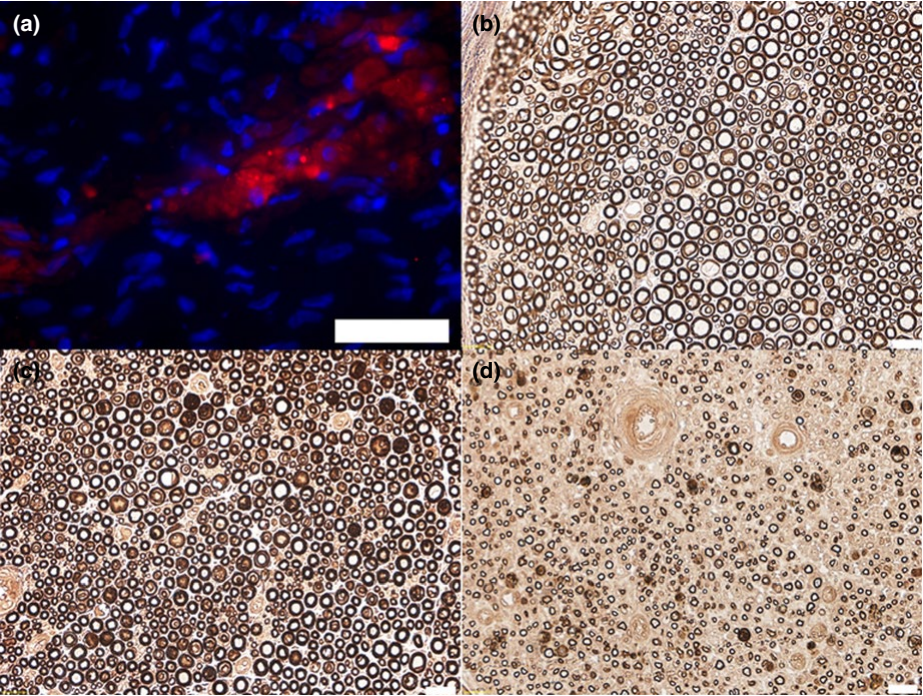
Representative images on PKH26‐labeled rASCs 4 weeks after injection (red) (a) Nuclei were stained with DAPI (4’‐6‐diamidino‐2‐phenylindole (in blue) Scale bar: 50 μm. Representative images of semithin osmium tetroxide‐p‐phenylenediamine‐stained cross sections (b‐d). Scale bar: 20 μm. (b) 40x magnification of the proximal part of a normal nerve (control group). (c) 40x magnification of a cross section of the proximal part 4 weeks after acute short trauma (d) 40x magnification of a cross section of the distal part 4 weeks after acute short trauma

**Figure 2:**
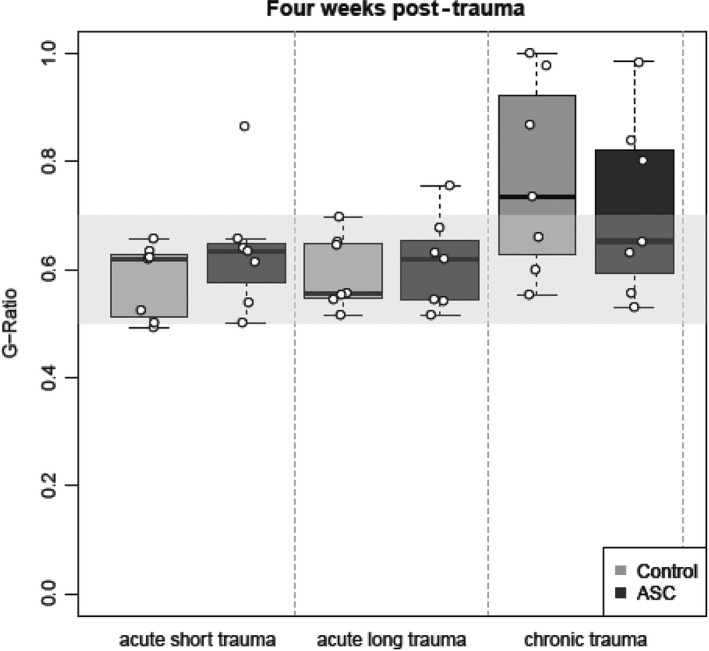
The G‐Ratio (ratio of the inner axonal diameter to the total outer diameter) of the traumatized nerve fiber 4 weeks after trauma. The closure the value is to one, the thinner the myelin of the traumatized nerve is, the closer it goes to zero, the thicker is the myelin. A healthy nerve has a G‐Ratio of between 0.5 and 0.7 (gray rectangle in the plot). Boxes represent first and third quartiles with the median central. The whiskers represent 5th and 95th percentiles, and the observed data are represented by the white dots

After 4 weeks, we successfully confirmed cell viability where PKH26‐labeled rASCs were arranged in densely arranged clusters as previously reported (Figure 1a) (Tremp et al., 2015). The G‐Ratio analyses showed after 2 weeks a higher ratio in group 1 (median 0.94, IQR 0.91–0.99) and group 3 (median 0.96, IQR 0.94–0.99) compared to group 2 (median 0.86, IQR 0.69–0.93), indicating a thinner myelin. After 4 weeks however, the ratio in the group 1 & 2 is similar and close to 0.6, indicating an optimal G‐Ratio similar to a healthy nerve (median 0.62, IQR 0.53–0.64 and median 0.59, IQR 0.54–0.65, respectively). In the chronic trauma group (group 3) however, the G‐ratio remains high (median 0.7, IQR 0.61–0.86), indicating a thinner myelin (Figure 2). In terms of regeneration of the G‐Ratio, the negative impact of the chronic trauma compared to the short trauma was statistically significant (effect of 0.064, 95%‐CI 0.014–0.0114, *p* = 0.01). Similarly, the median delta myelin in μm (difference between myelin thickness in the nerve proximal to the trauma (control) and the nerve distal to the trauma (traumatized) is similar in all groups after 2 weeks (group 1 median 1.32, IQR 1.21–1.41; group 2 median 1.19, IQR 0.94–1.34; group 3 median 1.39, IQR 1.23–1.49). Whereas after 4 weeks there is a higher median delta myelin in group 1 (median 0.56, IQR 0.22–0.84) compared to group 2 & 3 (median 0.47, IQR 0.22–0.57 and median 0.45, IQR 0.09–1.11, respectively).

### 3.2 Behavioral analysis

**Figure 3:**
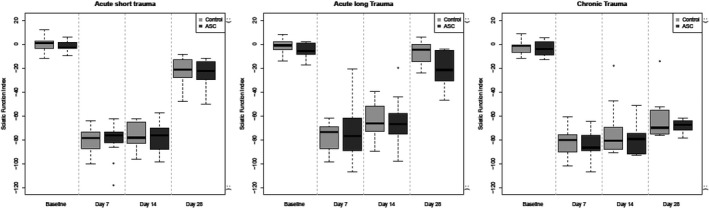
Boxplot of the sciatic functional index (SFI) over time stratified by treatment and trauma. Four weeks after trauma, the SFI in group 3 is significantly lower from the one in group 1

The SFI is decreased in all groups after the trauma was applied (clear decrease from day 0 to day 7) (Figure 3). From day 7–14 a recovery is visible in most of the animals (group 1 median −77.5, IQR −89 to −75 at day 7 and median −76, IQR −86 to −71 at day 14, group 2 median −85, IQR −97 to −69 at day 7 and median −66, IQR −81 to −55 at day 14, group 3 median −87, IQR −98 to −75 at day 7 and median −76, IQR −84 to −62 at day 14). The majority of the rats have an increase in the SFI values between these time points. But there are also some animals which are getting worse or remain as worse as during week one according to the SFI. Especially in group 1, the healing phase seems to be delayed compared to the other two groups.

From Day 14–28 the recovery is more progressed, all the rats could improve their SFI within this time period (group 1 median −22.23, IQR −29 to −14, group 2 median −22, IQR −31 to −5, group 3 median −68, IQR −72 to −64).

Multivariable regression analysis shows that there is no evidence for a treatment effect of ASCs in the data. However, the type of the trauma has an effect on the recovery and thus on the SFI and this effect changes over time. The rats with an acute long trauma are doing better than those with an acute short trauma at one and especially at 2 weeks after trauma (estimated effect on SFI 7.43, 95%‐CI −0.41‐15.26, *p* = 0.06 and 14.26, 95%‐CI 6.43–22.1, *p* < 0.001, respectively). There is no evidence that these two groups differ after 4 weeks, when the recovery is very advanced and almost all rats are back to normal or very close to an SFI of zero. Four weeks after trauma the SFI in group 3 is statistically significantly lower from the one in group 1 (estimated effect on SFI −42.02, 95%‐CI −52.76 to −31.29, *p* < 0.001).

### 3.3 MRI evaluation and muscle atrophy

**Figure 4:**
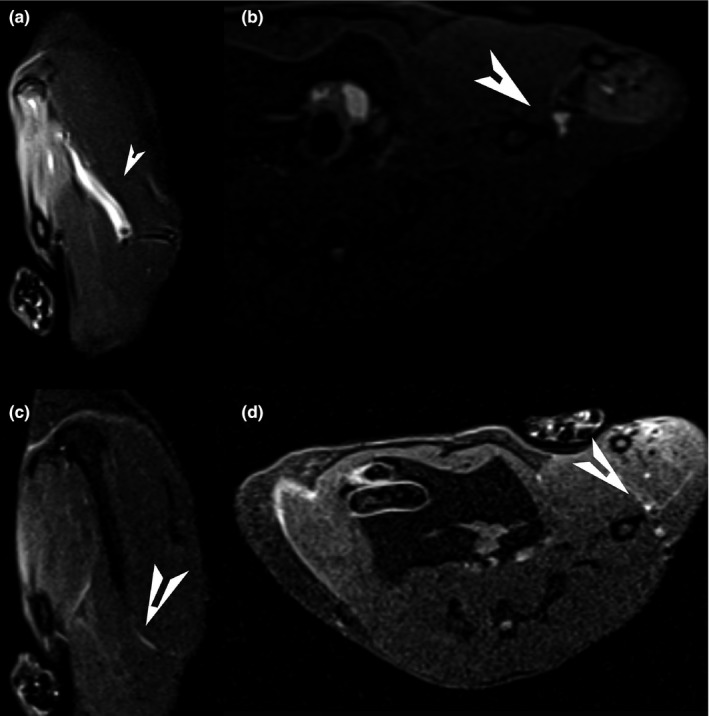
Representative MRI analysis. The crushed nerve is identified with the clip on MRI and surrounding edema around the nerve (a & b). In the acute short and long trauma group, less edema was found around the nerve (c & d)

**Figure 5:**
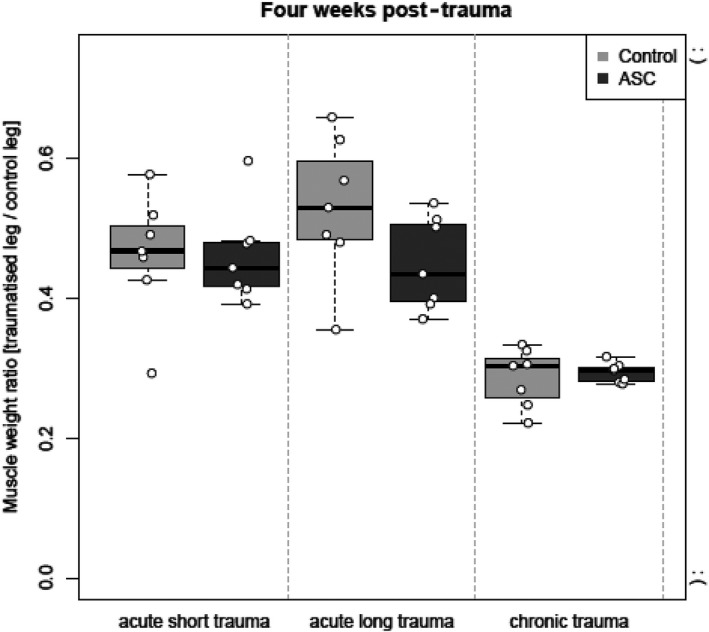
Muscle Weight Ratio (MWR) of the gastrocnemius muscle in the traumatized to the untraumatized leg 4 weeks post‐trauma. The closure the value is to zero, and the worse the traumatized leg is doing. The closure the value is to 1, and the better the traumatized leg is doing. The MWR after 4 weeks is better in group 1 & 2 compared with group 3. There was no significant difference between the rASCs group and the control group

We were able to identify the crushed nerve with the clip on MRI and surrounding edema around the nerve. In group 1 & 2, less edema was found around the nerve (Figure 4). Similar to functional analyses, the type of the trauma has an effect on the muscle volume after 2 and 4 weeks with better values in group 1 & 2. In the chronic trauma group the MWR was lower after 4 weeks (median 0.3, IQR 0.28–0.3 in group 3 vs. median 0.43, IQR 0.4–0.51 in group 2 and median 0.44, IQR 0.42–0.48 in group 1, respectively (Figure 5). There was no significant difference between the rASCs group and the control group.

## 4 DISCUSSION

In this study, we showed that the type of nerve compression has an effect on the recovery. Not surprisingly, the animals with a chronic trauma had the worst outcome compared to the rats with an acute short or long trauma, both functionally and by morphology/radiology. A higher relative myelin ratio and a lower delta myelin were found in the acute short and long trauma group compared to the chronic trauma group. The recovery seems to be faster in rats with an acute long trauma compared to rats with a short trauma, as evaluated by the SFI. Those results go in line with the muscle volume measurements, showing a higher MWR in the acute long trauma group. Moreover, we found in the rats with an acute long trauma a lower delta myelin, lower G‐Ratio and a lower difference in fractional anisotropy compared to the acute short trauma, indicating an overall better recovery of myelination.

Crush injuries can cause many different degrees of neural damage that can represent any of the class of the schemes described by Seddon or Sunderland (Sunderland, 1951). Crush injuries usually occur from an acute traumatic compression of the nerve from a blunt object that does not result in a complete transection of the nerve (Menorca, Fussell, & Elfar, 2013). In the recovery period, the axonal outgrowth becomes remyelinated by the resident Schwann cells (SCs). However, the myelination is usually much thinner than normal, with accordingly predictable electrical consequences (Kang, Zamorano, & Gupta, 2011).

The idea of using ASCs is the lack of supportive SCs or their inability to maintain a regenerative phenotype after peripheral nerve injuries (PNI) (Bhangra, Busuttil, Phillips, & Rahim, 2016). It has been suggested that nervous system tissue engineering technology can build Schwann cell scaffolds to overcome this and enhance the regenerative capacity of neurons following injury (Bhangra et al., 2016).

We recently successfully transplanted and compared human and autologous stem cells for peripheral nerve regeneration in a rat sciatic nerve injury model. Moreover, we were able to implement the clinical 3T MRI scanner to monitor the efficacy of cellular therapy over time (Tremp et al., 2015). In particular, differentiated ASCs could be a clinically translatable route toward new methods to enhance peripheral nerve repair (Kappos et al., 2015). Thus, we selected epineural rASC injection with the idea to boost peripheral nerve regeneration as it has be shown that ASCs enhance peripheral nerve regeneration in 86% of the experiments (Walocko, Khouri, Urbanchek, Levi, & Cederna, 2016). However, it has been suggested that it is possible for peripheral nerve fibers to spontaneously regenerate when continuity of the nerve is maintained during injury (Walocko et al., 2016), such as in our current study.

Alternative strategies and methods can be applied to enhance peripheral nerve regeneration using ASCs, for example, hydrogels as scaffolds and delivery systems, or intravenously, spreading throughout the body and get to their target organs by a homing response to the injured neurological tissue (Carballo‐Molina & Velasco, 2015; Cooney et al., 2016; Marconi et al., 2012; Zack‐Williams, Butler, & Kalaskar, 2015). In a recent study by Sowa et al., ASCs and SCs were transplanted with gelatin hydrogel tubes at the artificially blunted sciatic nerve lesion in mice. In their study, the transplantation of ASCs promoted regeneration of axons, formation of myelin, and restoration of denervation muscle atrophy to levels comparable to those achieved by SC transplantation. Importantly, ASCs survived for at least 4 weeks after transplantation without differentiating into SCs (Sowa et al., 2016).

In a recent study by Kilic et al. (2013), crush injury was performed, and inguinal adipose tissue with its vascular pedicle was mobilized and wrapped around the nerve lesion. In this study, maximum isometric tetanic force recovery was significantly greater in the autogenous fat graft group compared with the control (untreated nerve crush). Interestingly, while myelin thickness, total axon count, and nerve fiber density were significantly increased compared to the control group, no changes were noted in muscle mass. Thus, the effectiveness of whole avascular autogenous fat grafts on peripheral nerve regeneration needs to be studied in more detail (Kilic et al., 2013). In line with our results, we found no significant effect of ASCs on peripheral nerve regeneration.

Potentially, stromal vascular fraction (SVF) might be a more clinically translatable route toward new methods to enhance peripheral nerve repair. SVF can effectively induce new vessel formation through the dynamic reassembly of blood endothelial cells and could thus be applied to achieve therapeutic neovascularization (Kappos et al., 2015). Moreover, they are considered to be a fundamental requisite for nerve repair and it has the advantage of reduction in the interval from tissue collection until cell injection and simplicity of laboratory procedure, especially where a traumatic injury is dealt with (Kappos et al., 2015; Tremp et al., 2015).

It has been shown that ASCs express various neurotrophic factors, including nerve growth factor, brain‐derived neurotrophic factor, and glial‐derived neurotrophic factor (Kalbermatten, Schaakxs, Kingham, & Wiberg, 2011). However, full remyelination of regenerated axons may not occur due to simply insufficient stimulation by neuronal growth factors from SCs (Hughes, Kusner, & Kaminski, 2006). Our group has shown that rASC can be differentiated into cells resembling SCs, which promote neurite outgrowth in vitro (Kingham et al., 2007) and enhance peripheral nerve regeneration in vivo (di Summa et al., 2010). Although some trans‐differentiation of transplanted mesenchymal stem cells (MSCs) might occur in vivo, the exact mechanisms behind the neuroprotective and growth‐promoting effects of adult stem cells remain poorly investigated.

Some of the benefits elicited by transplanted cells might occur as a direct result of their production of neurotrophic factors. It is possible that the mechanisms promoting nerve regeneration rule out a process of differentiation of ASCs and rather suggest the relevance of a bystander effect, including the production of in situ molecules, which may modulate the local environment with the down‐regulation of inflammation and the promotion of axonal regeneration (Kang et al., 2011).

Moreover, it has been suggested that erythropoietin may have neuroprotective, and perhaps also neurotrophic roles in acute sciatic nerve crush injury (Elfar, Jacobson, Puzas, Rosier, & Zuscik, 2008; Grasso et al., 2007). Pan et al administered hyperbaric oxygen to a rat‐crushed sciatic nerve that was embedded in a fibrin glue rich of amniotic fluid mesenchymal stem cells. The authors found that the amniotic fluid mesenchymal stem cells/hyperbaric oxygen combined treatment showed the most beneficial effect (Pan et al., 2009). Interestingly, it has been also proposed that the application of electrical stimulation immediately after crush nerve injury in rats may promote nerve regeneration and accelerate remyelination (Alrashdan et al., 2010; Teodori et al., 2011; Vivo et al., 2008). Alternatively, a primary repair for crush injury of sensory and mixed nerves with biological tubulization (e.g., muscle‐vein‐combined graft, fibrin conduit (Tos, Battiston, Ciclamini, Geuna, & Artiaco, 2012; Pettersson, Kalbermatten, McGrath, & Novikova, 2010)). might restore the continuity of the nerve, avoiding secondary nerve grafting. Huang et al. (2016) suggested that the modulation of autophagy in PNI might be an effective pharmacological approach to promote nerve regeneration and reestablish motor function.

That being said, more studies are needed with a longer follow‐up including molecular analysis to demonstrate the potential therapeutic mechanism and to evaluate the optimal strategy with a multimodal approach for the treatment of peripheral acute and chronic nerve crush injury. In particular, the chronic nerve crush injury model has not been elucidated yet and needs further analyses.

To sum up, we successfully evaluated the regenerative effect of epineural injection of rASCs in three different settings of acute and chronic compression. Our results indicate that in the acute short and long peripheral nerve crush injuries, peripheral nerve repair is restored close to normal after 4 weeks. However, there were no significant differences in outcomes in the ASC‐treated groups and control groups.

## CONFLICT OF INTEREST

None declared.
